# Pennsylvania State Veterinary Medical Association

**Published:** 1893-10

**Authors:** 


### SOCIETY PROCEEDINGS.

*Pennsylvania State Veterinary Medical Association.*—The ninth annual meeting of the Pennsylvania State Veterinary Medical Association was held in the College of Physicians, Philadelphia, March 7th. The Association was called to order at 10:30 A. M., Dr. Hoskins in the chair.

On roll-call the following members answered to their names: Allen, Custer, Du Bois, Ferley, Formad, Gladfelter. Glass, Goentner. Harger, Hart, Bartholomew, Helmer, Hoffman, F. F. Hoskins, Keelor, Keil, Knight, Kooker, Lusson, Magee, Michener, J. C. Nunan, Pearson, Raynor, G. B. Raynor, Thos. B. Raynor, Jas. B. Raynor, John B. Reinhart, Ridge, Sallada, Schaufler, Sturge, Timberman, Weber, Webster and Zuill. As visitors: Drs. John Marshall, Dean Vet. Dept. Univ. of Pa.; W. B. Atkinson, of State Board of Health; Jas. B. McAnulty, John Adams, E Mayhew Michener, W. J. Hinds, M. E Conard, W. H. Fry, W. B. E Miller and a number of students Vet. Dept. Univ. of Pa.

The minutes of the semi-annual meeting were read, and, with a slight correction, adopted.

The President's report, reviewing the work of the Association for the past nine years, and congratulating the members on the volume of work performed, the high place attained and the bright promises of the future, was interesting and instructive.

The election of officers resulted in the selection of President, W. Horace Hoskins; first Vice-President, Dr. Leonard Pearson; second Vice-President. Dr. Jas. B. Rayner; third Vice-President, Dr. Z. S. Keil; Recording Secretary, Dr. Robt. Gladfelter; Corresponding Secretary, Dr. W. H. Ridge; Treasurer, Dr. John R. Hart. Board of Trustees: Dr. S. J. J. Harger, Jas. Sallade, J. C. Michener, W. S. Kooker, Leonard Pearson.

The following list of applicants were then read and referred to the Board of Trustees: R. A. Dunn, D.V.S.; A. V. C.; Titusville, Pa.; W. H. Ridge, voucher. A. O. Cawley, D.V.S.; A. V C.; Milton, Pa.; W. H. Ridge. voucher. B. L. J. Bear, V.M.D.; V. D. Univ. of Pa.; York, York Co., Pa.; W. H. Ridge, voucher. Geo. A. Smith, V.M D.; V. D. Univ. of Pa.; 16th and Bainbridge Streets. Phila., Pa.; W. H. Ridge and Leonard Pearson, vouchers. E. Mayhew Michener, V.M.D.; V. D. Univ. of Pa.; Colmar, Pa.; W. H. Ridge, voucher. Chas. A. Dohan, V.M.D.; V. D. Univ. of Pa.; Darling, Delaware Co., Pa.; W. H. Ridge, voucher. Chas. M. Cullen, V.M.D.; V. D. Univ. of Pa.; 4317 Lancaster Avenue, Philadelphia, Pa.; W. H Ridge, voucher. John J. Maher, V.M.D.; V. D. Univ. of Pa.; 1514 Marshall Street, Philadelphia, Pa.; W. H. Ridge, voucher. M. E. Conard, V.M.D.; V. D. Univ. of Pa.; West Grove, Pa.; W. H. Ridge, voucher. Horace P. Keely, V.M.D.; V. D. Univ. of Pa.; Schwenksville, Pa.; W. H. Ridge, voucher. David Martin, V.S.; McKeesport, Pa.; Jas. A. Waugh voucher. J. Heckenberger, V.S.; Ontario; Catasauqua, Pa.; W. H. Ridge and John C. Foelker, vouchers. Wm. A. Heckenberger, V.S.; Ontario; Catasauqua, Pa.; John C. Foelker and W. S. Kooker, vouchers. Harry D. Entrikin, V.M.D.; V. D. Univ. of Pa.; Kennett Square, Pa.; W. H. Ridge, voucher. S. D. Larzelere, V.M.D.;

V. D. Univ. of Pa.; Jenkintown, Pa.; W. H. Ridge, voucher. C. Lintz, V.M.D.; V. D. Univ. of Pa.; Chester, Pa.; W. H. Ridge, voucher. F. M. Kain, D.V.S.; A.V.C.; York, York Co., Pa.; W. H. Ridge, voucher. Frank L. Smith, V.M.D.; V. D. Univ. of Pa.; 2027 North 13th Street, Philadelphia, Pa.; John R. Hart and W. H. Ridge, vouchers. Guildin R. Hartman. V.M.D.; V. D. Univ. of Pa.; 2130 North 4th Street, Philadelphia, Pa.; John R. Hart and W. H. Ridge, vouchers. Dr. J. J. McCarthey, V.S.; Ontario Vet. College; vouchers, J W. Sallada and W. H. Fry; Pine Grove Mills, Pa. Joseph Houldsworth. V.M.D.; V. D. Univ. of Pa., Philadelphia, Pa.; voucher, W. H. Ridge. Wm. J. Tomlinson, D.V.S.; Am. Vet. College; Williamsport, Pa.; vouchers, W. Horace Hoskins and Robt. Gladfelter. Jas T. McAnulty, V.S.; Philadelphia, Pa.; voucher, W. H. Hoskins. W. B. E. Miller, D.V.S.; Am. Vet. College; Philadelphia, Pa.; vouchers, W. H. Hoskins and Thos. B. Raynor.

A recess was then taken, that the Board of Trustees might convene to examine the applicants.

On reconvening, the Board recommended for favorable action: Drs. Dunn, Bear, Smith, Geo A. Michener, Dohan, Cullen, Maher, Conard, Keely, J. Heckenberger, Wm. A. Heckenberger, Entrikin, Larzelere, Lintz, Kain, Frank L. Smith, Hartman, Houldsworth, Miller and McCarthey. The Board unfavorably recommends: Drs. Fry, McAnulty and Martin; and laid over for further consideration the applications of Drs. Tomlinson, Cawley and Fox.

A motion to dispense with by-laws and elect by acclimation was carried, and those favorably recommended were, on motion, declared elected.

The President introduced the new members who were present.

The Corresponding Secretary's report followed, containing much information relative to the work of the past six months; calling brief attention to the new local veterinary societies in the Wyoming Valley; and, among many other valuable points, jogging the faulty memories of many of the members for their negligence in not replying to and acknowledging communications from the Secretary's office.

The Treasurer's report showed exceedingly heavy expenditures for the year; and though the balance was on the wrong side, a large income from the members had been received, and never in the history of the Association were there so few delinquents as at present.

Under unfinished business, the following amendments were adopted:

1st. That on and after the year of 1893 the dues of this association shall be two dollars, to be made in semi-annual payments.

2d. Any member in arrears for his initiation fees or dues for a period of eighteen months, shall receive two quarterly notices of said arrearage, and in failing to liquidate the same shall be reported to the Association by the Treasurer for expulsion.

Under new business, Dr. S. J. J. Harger proposed that this association place itself in sympathy and work with the National Association in the proposed International Congress. After much discussion a motion prevailed that the chair appoint a committee of nine to represent this association in completing arrangements for our members at Chicago, performing any duties assigned our organization, or preparing to fulfil any part of the program toward making successful this coming Congress

The subject of participating in the proper reception of foreign visitors, delegates or members of the profession from abroad, in conjunction with committees from other States and local associations was suggested by Dr. Pearson, and after considerable discussion it was moved and adopted that the chair appoint a committee of nine to receive and entertain, during their trip through our State, all foreign delegates to our Congress.

Under the above resolutions the President appointed the following committees:

*Committee on International Meeting.*—Dr. S. J. J. Harger (Chairman), W. S. Kooker, L. O. Lusson, Alex. Glass, Jas. B. Rayner, C. T. Goentner, J. C. Foelker, Robt. Formad and J. B. Irons.

*Committee on Entertainment of Foreign Delegates.*—Drs. Leonard Pearson (Chairman), Thos. B. Raynor, W. L. Zuill, W. B. E. Miller, J. Timberman, J. Helmer, Jas. A Waugh, J. C. McNeil, Chas. Schaufler.

Reports of committees being in order, the first one called being on legislation. Chairman Kooker reported results of prosecutions at Washing, Pa., under the veterinary act, together with the proposed act to secure a pure, wholesome and unadulterated milk supply, and to provide for licensing milk producers and milk venders, and for the appointment of Milk and Dairy Inspectors in the State, together with several amendments to existing acts pertaining to the milk and meat supply of our commonwealth. (*Vide, fol.* 266.)

Chairman Weber, of the Committee on Intelligence and Education, then read one of the most interesting, instructive, incisive and important ever produced for the association to consider, embodying many special points and suggested avenues of labor for the profession to enter upon. (*Vide, fol.* 266.)

Chairman Harger, of the Committee on Sanitary Science and Policy, presented a brief report, noting many new discoveries and referring to the present experiments in detecting glanders and tuberculosis and the growing theory in the contagiousness of tetanus.

The Committee of Arrangements then announced that the Philadelphia veterinarians had procured sufficient seats at the Broad Street Theatre for all those in attendance, and were very anxious to have all present. After some minor business the meeting adjourned until 10 A. M. the following day.

